# Effects of babassu nut oil on ischemia/reperfusion-induced leukocyte adhesion and macromolecular leakage in the microcirculation: Observation in the hamster cheek pouch

**DOI:** 10.1186/1476-511X-11-158

**Published:** 2012-11-16

**Authors:** Maria do Carmo L Barbosa, Eliete Bouskela, Fátima ZGA Cyrino, Ana Paula S Azevedo, Maria Célia P Costa, Maria das Graças C de Souza, Debora S Santos, Felipe L Barbosa, Luiz Felipe A Guerra, Maria do Desterro SB Nascimento

**Affiliations:** 1 Laboratory of Basic and Applied Immunology and Laboratory of Imunophisiology, Center for Biological and Health Sciences, Federal University of Maranhão, Bacanga Campus, Av. dos Portugueses s/n, Bloco 3, sala 3A, 65085-580, São Luís MA, Brazil; 2 Laboratory for Clinical and Experimental Research on Vascular Biology (BioVasc), Reitor Haroldo Lisboa da Cunha Complex, Rio de Janeiro State University, Rua São Francisco Xavier, 524, Ground Floor, 20550-013, Rio de Janeiro, RJ, Brazil; 3 Laboratory of Macromolecular and Natural Products, Department of Chemistry and Biology, Maranhão State University, Center of Education, Exact Sciences, and Natural Sciences, Paulo VI Campus, Caixa Postal 09, 65055-970, São Luís, MA, Brazil

**Keywords:** Orbignya phalerata, Arecaceae, Babassu oil, Ischemia/reperfusion, Vascular permeability

## Abstract

**Background:**

The babassu palm tree is native to Brazil and is most densely distributed in the Cocais region of the state of Maranhão, in northeastern Brazil. In addition to the industrial use of refined babassu oil, the milk, the unrefined oil and the nuts *in natura* are used by families from several communities of African descendants as one of the principal sources of food energy. The objective of this study was to evaluate the effects of babassu oil on microvascular permeability and leukocyte-endothelial interactions induced by ischemia/reperfusion using the hamster cheek pouch microcirculation as experimental model.

**Methods:**

Twice a day for 14 days, male hamsters received unrefined babassu oil (0.02 ml/dose [BO-2 group], 0.06 ml/dose [BO-6 group], 0.18 ml/dose [BO-18 group]) or mineral oil (0.18 ml/dose [MO group]). Observations were made in the cheek pouch and macromolecular permeability increase induced by ischemia/reperfusion (I/R) or topical application of histamine, as well as leukocyte-endothelial interaction after I/R were evaluated.

**Results:**

The mean value of I/R-induced microvascular leakage, determined during reperfusion, was significantly lower in the BO-6 and BO-18 groups than in the MO one (*P* < 0.001). In addition, histamine-induced increase of microvascular permeability was significantly less pronounced in BO groups compared to MO one. No significant differences among groups in terms of leukocyte adhesion, concentrations of tumor necrosis factor alpha, interleukin 1, and interleukin 6 were found.

**Conclusions:**

Our findings suggest that unrefined babassu oil reduced microvascular leakage and protected against histamine-induced effects in postcapillary venules and highlights that these almost unexploited nut and its oil might be secure sources of food energy.

## Background

The babassu palm tree is native to Brazil and is found in the deciduous forests of eastern Amazonia, especially in the state of Maranhão. The predominant species is *Orbignya phalerata* Mart. (Arecaceae), which has been widely studied [1-3]. Although the genus *Orbignya* is found in other countries of the Americas, the concentration of these species is believed to be highest in the Cocais region of Maranhão [4,5].

Babassu oil extracted from the nut is very rich in triacylglycerols, small amounts of free fatty acids, phospholipids, pigments, sterols and tocopherols [6]. There is a wide variety of fatty acids including lauric acid (44%), oleic acid (17%), myristic acid (17%), palmitic acid (8%), capric acid (6%), caprylic acid (5%), stearic acid (4.5%), and linoleic acid (2%) [7,8].

Each fruit contains 3 to 6 nuts that are collected manually in a traditional home subsistence system [9]. In addition to its industrial use as refined edible oil [10], the milk and the unrefined oil of the babassu nut are used for cooking by families from several communities of African descendants as one of the principal sources of food energy. Nuts are also eaten *in natura*[9,11]*.*

Nowadays plant seeds constitute new oil sources and there is a worldwide interest for exploitation from natural resources. Unlike other edible oils, little is known about the possible damages or benefits of the dietary use of the babassu nut and its products for human health. On the other hand, fish oil and olive oil have been reported to protect against cardiovascular disease [12,13] and to improve clinical indicators of disease activity in rheumatoid arthritis patients [14].

The reputation of olive oil as a healthy product is more likely due to the presence of numerous minor elements contained in it like biophenols, some triterpene alcohols, phytosterols, squalene and tocopherols [15,16].

Phytosterols and tocopherols are also present in babassu oil [6]. Plant sterols are natural dietary components fundamental for cell membranes in both plants and animals with serum-lowering proprieties [17]. Tocopherols are considered to be the most effective lipid phase natural antioxidant which plays an important role in cellular signaling, especially in relation to protein kinase C [18].

In the present study, we used the hamster cheek pouch preparation in order to evaluate, for the first time, effects that unrefined babassu oil has on ischemia /reperfusion induced microvascular permeability and leukocyte-endothelial interactions.

## Material and methods

### Animals

We used 39 male golden hamsters (*Mesocricetus auratus*; Paulinea, São Paulo, Brazil), weighing 122–146 g, at 7–10 weeks of age. Animals were housed in a temperature- and humidity-controlled environment, on a 12/12-h light/dark cycle, with *ad libitum* access to water and autoclaved standard rodent chow (Nuvital; Nuvilab, Curitiba, Brazil), at the State University of Rio de Janeiro, Laboratory of Clinical and Experimental Research in Vascular Biology, Rio de Janeiro, Brazil.

Hamsters were divided into four groups of animals (*n* = 9/group), treated twice a day for 14 days, either with mineral oil — 0.18 ml/dose (MO group) — or with unrefined babassu oil — 0.02 ml/dose (BO-2 group), 0.06 ml/dose (BO-6 group); and 0.18 ml/dose (BO-18 group). Those four groups were further subdivided: 6 animals from each group were used for analysis of permeability and leukocyte adhesion; and 3 others from each group were used to determine levels of interleukin (IL)-1, IL-6, and tumor necrosis factor alpha (TNF-α). Another group of untreated animals (receiving neither mineral oil nor babassu oil, *n* = 3) was created as an additional control for the determination of cytokine levels. Sterile mineral oil was used as negative control because it is considered safe [19] and it has reduced absorption in the gastrointestinal tract [20].

All procedures were approved by the State University of Rio de Janeiro Committee for Animal Experimentation (Protocol no. 215/2007) and were conducted in accordance with international standards for biomedical research involving animals, as well as with those established by the Brazilian College of Animal Experimentation.

### Obtaining the babassu oil

Kernels were collected from babassu trees in the municipality of Codó, located in the state of Maranhão, and the oil was extracted at the Chemistry Laboratory of Maranhão State University, in the city of São Luís. We have performed continuous extraction using a Soxhlet extractor (Foss Tecator Soxtec HT 6; Fisher Scientific, Pittsburgh, PA, USA) and a heater (Tecnal, Piracicaba, Brazil) that had been preheated in an incubator for 60 min at 150°C. The solvent used was hexane [21-23].

### Intravital microscopy studies

On the day of the experiment anesthesia was induced by intraperitoneal injection of sodium pentobarbital −0.02ml/100g/body weight (Pentobarbital sodique, Sanofi, Paris, France, 60mg/ml) and maintained with intravenous injection of α-chloralose (2.5% solution) through a femoral vein catheter. To facilitate spontaneous breathing, a tracheal cannula (PE 190, Becton Dickinson, and Sparks, MD, USA) was inserted. Body temperature was maintained at 37°C with a heating pad and monitored with a rectal thermistor. The preparation of the cheek pouch for intravital microscopy has been previously described [24-26]. In brief, the cheek pouch was everted and mounted on a microscope stage. An area of approximately 1 cm^2^ was prepared for intravital microscopy of macromolecular permeability and leukocyte adhesion. The cheek pouch was continuously superfused with warm (36.5°C) HEPES bicarbonate buffered saline solution gassed with 95% N_2_5% CO_2_ to maintain a pH of 7.4 and low oxygen tension. At 30 min after completion of the preparation phase, each hamster received an intravenous injection (25mg/100g body weight) of fluorescein isothiocyanate (FITC)-labeled dextran molecular weight, 150 000, TdB Consultancy, Uppsala, Sweden) as a macromolecular tracer. Total ischemia was achieved with an inflatable cuff placed around the neck of the everted pouch [24] maintained for 30 min. Microvascular permeability to large molecules was quantified by counting the number of sites of fluorescent plasma extravasation (leaks), visualized by tracer fluorescence in postcapillary venules [24,25]. Preparations with less than 10 leak sites 30 min after FITC-labeled dextran injection were accepted for experiments. At 60 min after reperfusion, histamine (5^×^10ˉ^6^ M) (Sigma, St. Louis, MO, USA) was applied topically for 5 min.The number of leaks was counted at 2, 5, 10 and 15 min after the application of the histamine. The number of leaks at 10 min after reperfusion and 5 min after histamine application were used for statistical calculations.

### Cytokine measurements

Blood samples were collected by cardiac puncture while animals were maintained under anesthesia. The serum was stored (−80°C) to determine levels of IL-1, IL-6, and TNF-α using high-sensitivity ELISA kit (R&D System, Minneapolis, MN, USA),according to the protocol specified by the manufacturer.

### Statistical analysis

Statistical analyses were performed with the STATA program, version 9.0 for Windows (Stata Corp, College Station, TX, USA). Data are expressed as mean and standard deviation. In comparisons among groups and among time points, we have used analysis of variance (ANOVA) followed by Tukey’s *post hoc* test. The homogeneity of variances was assessed by Levene’s test. Data were graphed by box-plot. For all statistical tests, values of *P* < 0.05 were considered statistically significant.

## Results

### Intravital microscopy studies

**Figure 1:**
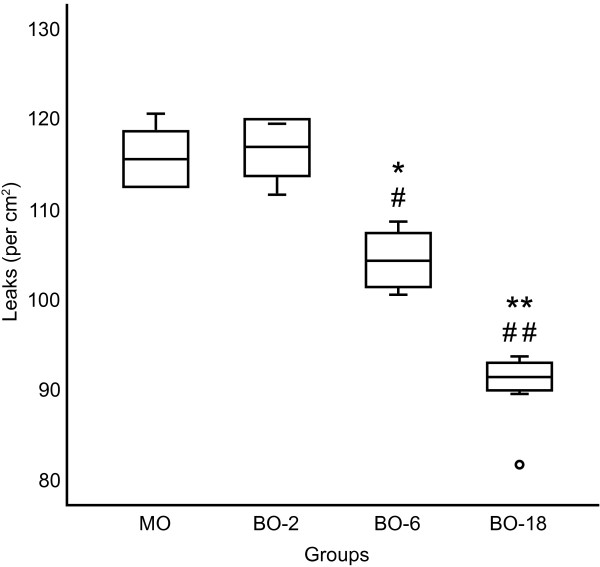
**Ischemia-induced vascular leakage (leaks/cm**^**2**^**), at 10 min after the onset of reperfusion, in groups of hamsters treated twice a day for 14 days with 0.18 ml of mineral oil (MO), 0.02 ml (BO-2), 0.06 ml (BO-6) or 0.18 ml (BO-18) of unrefined babassu oil.** The lines represent the mean (center line) and the standard deviation (top and bottom line). The whiskers represent the maximum and minimum values. Analysis of variance ANOVA and Tukey’s post hoc test: * P<0.01, **P<0.001 compared with MO and #P<0.01 ##P<0.001 compared with BO-2.

Treated hamsters accepted the oral administration of mineral or babassu oils and responded to the ischemia/reperfusion. The number of ischemia-induced microvascular leaks during reperfusion was significantly lower in the BO-6 and BO-18 groups than in the MO and BO-2 groups (Figure 1).

**Figure 2:**
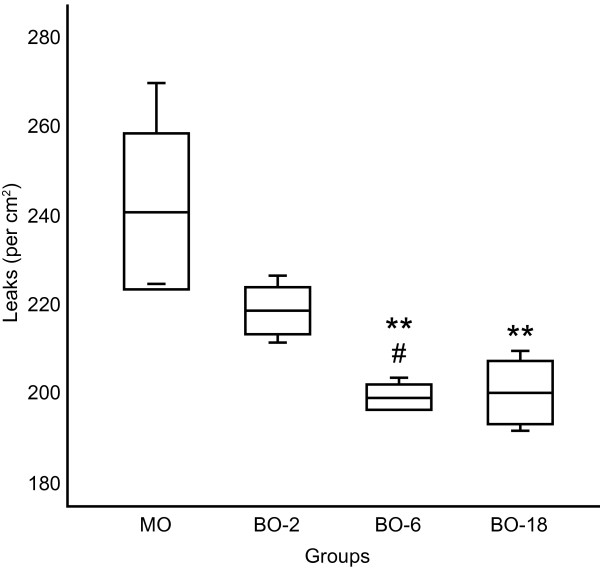
**Ischemia-induced vascular leakage (leaks/cm**^**2**^**), at 5 min after histamine application, in groups of hamsters treated twice a day for 14 days with the following: 0.18 ml of mineral oil (MO); 0.02 ml (BO-2); 0.06 ml (BO-6) or 0.18 ml of unrefined babassu oil (BO-18).** The lines represent the mean (center line) and the standard deviation (top and bottom line). The whiskers represent the maximum and minimum values. Analysis of variance ANOVA and Tukey’s post hoc tests: **P<0.001 compared with MO and #P<0.01 compared with BO-2.

The histamine-induced increase in microvascular permeability was significantly less pronounced in BO-6 and BO-18 groups treated with babassu oil (Figure 2).

### Leukocyte adhesion and cytokine levels

At both time points evaluated, the degree of leukocyte adhesion was significantly lower in BO-2 group than in BO-18 group (*P* < 0.05).

We found no significant differences among groups in terms of leukocyte adhesion, concentrations of tumor necrosis factor alpha, interleukin 1, and interleukin 6.

## Discussion

Atherosclerosis is considered to be an inflammatory disease and the endothelium plays a key role in this process [26]. It produces several vasoactive compounds like vasodilators [endothelium-derived relaxing factors (EDRFs)], such as nitric oxide (NO), prostacyclin and endothelium-derived hyperpolarizing factor (EDHF) and vasoconstricitors [endothelium-derived contracting factors (EDCFs)], such as angiotensin II, endothelin, and thromboxane A2/prostaglandin H_2_ and superoxide anions/isoprostane [27].

It has been shown that among the major known risk factors related to cardiovascular diseases, eating incorrectly has received special attention [28]. A low fat, hight-fiber diet, rich in dietary antioxidants from fruits, vegetables and whole grains is recommended to help reduce cardiovascular risk in the general population [29]. In this regard, the lipids are the most studied macronutrients concerning on the effects of dietary components on inflammation [30].

The oil extracted from the babassu nut is a complementary part of the diet of several indigenous [31] and babassu-nut breakers communities and is one of the principal sources of food energy [10,32].

In the present study, despite the presence of saturated fat acid, we have observed that treating male hamsters with unrefined babassu oil decreased ischemia-induced macromolecular leakage in postcapillary venules and protected against histamine-induced increase in microvascular permeability. However the degree of leukocyte adhesion was significantly lower only in the BO-2 group, compared to BO-18 one, data that need further investigations. It is important to emphasize that the histamine effect is not dependent on neutrophil inhibition [33,34].

Unrefined babassu oil contains minor components such as sterols and tocopherols [6] and oleic acid [7,8] which have been investigated as responsibly for protective effect on the microcirculation.

Previous studies, comparing activities of α-tocopherol and shark cartilage given orally to hamsters have shown that both inhibited macromolecular permeability increase after ischemia/reperfusion but only α-tocopherol was effective in decreasing the macromolecular permeability increase induced by histamine.

Several different studies of I/R-induced plasma leakage in the hamster cheek pouch concluded that at least two reactive oxygen species are involved such as superoxide (O_2_) and nitric oxide (NO). Superoxide dismutase (EC-SOD and CuZn-SOD) inhibited I/R-induced plasma leakage [35] and the nitric oxide synthetase inhibitor L-NA inhibited I/R-induced plasma leakage and leucocyte adherence to post capillary venules [36].

Vitamin E is considered the most important lipid-soluble antioxidant and lipid-rich plant products and vegetables are the main natural sources of it [37]. Humans absorb all forms of vitamin E, but the body maintains only α-tocopherol [38], incorporated into cellular membranes in which it effectively inhibits lipid peroxidation [39].

Other compounds present in the babassu oil are phytosterols [6], related to cholesterol-lowering properties of some oils from natural plant sources, but the exact mechanisms is not fully understood [40].

The babassu nut is an important source of lauric acid but it has 17% of oleic acid. Baer and co-workers demonstrated that ingestion of 8% oleic acid could inhibit the inflammatory process [41]. Whether or not the oleic acid plays a role in the protective effect observed in our experiments needs further investigation. Olive oil contains large amounts of oleic acid which is a ω-9 monounsaturated fatty acid converted to eicosatrienoic (ETA). ETA is converted to Leukotriene A_3_ (LTA_3_), which is a potent inhibitor of leukotriene B_4_ synthesis [42].

Given the limitations of our study and the fact that, to our knowledge, this is the first study to investigate effects of babassu oil in a model of ischemia-reperfusion injury, further studies will also be needed in order to corroborate our data and to justify the use of babassu oil as a source of food energy.

## Conclusions

Unrefined babassu oil given orally to male hamsters decreased ischemia-induced macromolecular leakage in postcapillary venules, and protected against a histamine-induced increase in microvascular permeability. Our results suggest that this almost unexploited nut and its oil might be secure sources of food energy.

## Competing interests

The authors declare that they have no competing interests.

## Authors’ contributions

MCLB conceived drafting, performed experimental work, interpretation and discussion of the results and wrote the paper. FZGA, MGCS participate of the experimental work analysis and interpretation. DSS, FLB, LFAG, MCPS, APSA participate in the acquisition of data, conception and interpretation. EB, MDSBN, participate in the conceiving drafting, orientation and revision the manuscript critically for important intellectual content. All authors read and approved the final manuscript.

## Authors’ information

Maria do Carmo Barbosa Lacerda, professor and researcher of the Federal University of Maranhão, Brazil, Master of Science in physiology and completing a doctoral thesis by the Biotechnology Network of Northeastern Brazil. She studies the health of black communities "Quilombolas" and babassu nut in the diet of these communities.

Eliete Bouskela professor and researcher, director of the Laboratory for Clinical and Experimental Research on Vascular Biology (BioVasc), of the Rio de Janeiro State University, Brazil. Fellow by the National Research Council (CNPq). She develops researcher concerning on regulation of microvascular reactivity in obesity, insulin resistance, septic shock, hemorrhagic stroke and cardiovascular risk detection using non-invasive methods.

Maria do Desterro Soares Brandão Nascimento professor and researcher, chief of the Laboratory of Basic and Applied Immunology of the Federal University of Maranhão, Brazil. She also studies the health of black communities "Quilombolas" and babassu nut in the diet of these communities.

Fátima ZGACyrino, biomedical and researcher of the Laboratory for Clinical and Experimental Research on Vascular Biology (BioVasc), of the Rio de Janeiro State University, Brazil. She develops the same research line of Dr Eliete Bouskela.

Ana Paula S.Azevedo, professor and researcher at the Laboratory of Imunophisiology of the Federal University of Maranhão, Brazil. She studies the Ethnopharmacology of regional plants, especially the babassu palm trees.

Maria Célia P Costa, professor and researcher, PhD in Chemistry, chief of the Laboratory of Macromolecular and Natural Products, Department of Chemistry and Biology, Maranhão State University, Brazil.

Maria das Graças C. de Souza, biologist and researcher the Laboratory for Clinical and Experimental Research on Vascular Biology (BioVasc), of the Rio de Janeiro State University, Brazil. She develops the same research line ofDr Eliete Bouskela.

Debora S Santos, Chemistry and research fellow of the Laboratory of Macromolecular and Natural Products, Department of Chemistry and Biology, Maranhão State University, Brazil.

Felipe L Barbosa and Luiz Felipe A Guerra, undergraduate researchers participants by FAPEMA- Foundation for the Support of Research and Technological Development in the State of Maranhão.
